# Enhanced surveillance and data feedback loop associated with improved malaria data in Lusaka, Zambia

**DOI:** 10.1186/s12936-015-0735-y

**Published:** 2015-05-29

**Authors:** Zunda Chisha, David A. Larsen, Matthew Burns, John M. Miller, Jacob Chirwa, Clara Mbwili, Daniel J. Bridges, Mulakwa Kamuliwo, Moonga Hawela, Kathrine R. Tan, Allen S. Craig, Anna M. Winters

**Affiliations:** Akros, Cresta Golfview Grounds, Great East Road, Unit 5, Lusaka, Zambia; Department of Public Health, Food Studies and Nutrition, Syracuse University, Syracuse, NY USA; PATH Malaria Control and Elimination Partnership in Africa (MACEPA), National Malaria Control Centre, Chainama Hospital College Grounds, Great East Road, Lusaka, Zambia; Department of Public Health and Research, National Malaria Control Centre, Ministry of Health, P.O. Box 32509, Lusaka, Zambia; Lusaka District Health Management Team, Ministry of Health, Lusaka, Zambia; Malaria Branch and US President’s Malaria Initiative, Centers for Disease Control and Prevention, Atlanta, GA USA; University of Montana School of Public and Community Health Sciences, Missoula, MO USA

**Keywords:** Surveillance, Malaria, Case management, Elimination, DHIS2, Zambia

## Abstract

**Background:**

Accurate and timely malaria data are crucial to monitor the progress towards and attainment of elimination. Lusaka, the capital city of Zambia, has reported very low malaria prevalence in Malaria Indicator Surveys. Issues of low malaria testing rates, high numbers of unconfirmed malaria cases and over consumption of anti-malarials were common at clinics within Lusaka, however. The Government of Zambia (GRZ) and its partners sought to address these issues through an enhanced surveillance and feedback programme at clinic level.

**Methods:**

The enhanced malaria surveillance programme began in 2011 to verify trends in reported malaria, as well as to implement a data feedback loop to improve data uptake, use, and quality. A process of monthly data collection and provision of feedback was implemented within all GRZ health clinics in Lusaka District. During clinic visits, clinic registers were accessed to record the number of reported malaria cases, malaria test positivity rate, malaria testing rate, and proportion of total suspected malaria that was confirmed with a diagnostic test.

**Results and discussion:**

Following the enhanced surveillance programme, the odds of receiving a diagnostic test for a suspected malaria case increased (OR = 1.54, 95 % CI = 0.96–2.49) followed by an upward monthly trend (OR = 1.05, 95 % CI = 1.01–1.09). The odds of a reported malaria case being diagnostically confirmed also increased monthly (1.09, 95 % CI 1.04–1.15). After an initial 140 % increase (95 % CI = 91–183 %), costs fell by 11 % each month (95 % CI = 5.7–10.9 %). Although the mean testing rate increased from 18.9 to 64.4 % over the time period, the proportion of reported malaria unconfirmed by diagnostic remained high at 76 %.

**Conclusions:**

Enhanced surveillance and implementation of a data feedback loop have substantially increased malaria testing rates and decreased the number of unconfirmed malaria cases and courses of ACT consumed in Lusaka District within just two years. Continued support of enhanced surveillance in Lusaka as well as national scale-up of the system is recommended to reinforce good case management and to ensure timely, reliable data are available to guide targeting of limited malaria prevention and control resources in Zambia.

## Background

Data-driven decision-making amongst national malaria control programmes in Africa is critical for the efficient use of resources among an often increasingly stratified malaria burden. Despite the need for improved information, routine malaria surveillance throughout sub-Saharan Africa is known to have many challenges including underrepresentation of the true burden of malaria circulating in communities [1–3], as well as lacking quality and timely data reporting. As malaria control programmes pursue malaria elimination, timely, reliable data becomes crucial to respond to potential resurgence and to target foci of malaria transmission with appropriate interventions [4, 5].

Improvements in Health Management Information Systems (HMIS) are needed in order to provide quality data necessary for a responsive malaria programme [6, 7]. However, national malaria control programme data are often from population-based surveys conducted at intervals of two years or more, rather than relying upon surveillance data from continuously operating health information systems [8, 9]. Scaling up and integrating the reporting of diagnostic confirmations of malaria cases has the potential to significantly reduce malaria reporting and unnecessary anti-malarial treatment administration, especially in areas with variable malaria transmission patterns [10].

The HMIS in Zambia collects and monitors health-related indicators through paper-based reporting systems collected on a monthly basis. Although collecting accurate and timely data has been a goal of the Government of Zambia (GRZ) since the inception of the HMIS, operational challenges remain. These include inadequate support for training of especially new facility staff on reporting standards and processes; infrequent supervision, especially amongst more rural, and distant facilities; and insufficient data validation across multiple reporting forms [11]. The lack of consistent feedback to health facilities regarding data quality and trends has previously been identified in an assessment of the HMIS system [11]. Furthermore, the time from a patient encounter at a clinic to receipt of those data at the central level can take weeks or even months.

In response to some of these challenges, the Ministry of Health (MOH) recently began implementing the District Health Information System 2 (DHIS2), a centralized, web-based aggregate reporting system at national, provincial, and district level to expedite data access and reporting. The DHIS2 data flow model improves access to information and timeliness from the point of data entry. It further allows greater functionality for reporting, feedback, and communication among system users. As the basis for aggregate reporting, DHIS2 was also used by the National Malaria Control Programme (NMCP) for data reporting from facility and community levels in areas of low malaria burden in support of the National Malaria Strategic Plan goal to bring areas closer to malaria elimination [12].

To further improve the accuracy and timeliness of malaria data and to understand the true malaria burden within all GRZ health facilities within Lusaka District, the NMCP, and its partners initiated an enhanced surveillance programme in 2010. This paper examines trends in malaria indicators before and after the enhanced surveillance programme, and assesses the benefits these investments had on reducing the levels of reported malaria in the district.

## Methods

### Study site

Lusaka District, located in the southern area of the central plateau of Zambia and home to the capital city Lusaka with an estimated population of 1.7 million, is the most urbanized area of the country. Lusaka District alone represents approximately 14 % of the national population and therefore resource allocation for malaria control efforts for Lusaka greatly affects national planning efforts. Recent survey reports suggest that Lusaka Province, including areas in Lusaka District are amongst the lowest malaria prevalence areas in Zambia, with less than 1 % malaria parasite prevalence reported in children under the age of 5 over the past 8 years [13–16]. Although indoor residual spray campaigns have been conducted since 2003, the distribution of long lasting insecticide treated nets (through mass distributions, at antenatal clinics, and at clinics for children under 5) as well as the use of artemisinin combination therapy (ACT) for clinical management of malaria are considered by some to be major contributing factors to low malaria prevalence [17]. Low malaria prevalence in Lusaka is also likely an outcome of the rapid rate of urbanization and high altitude [18, 19]. Routine HMIS data in Lusaka, Zambia reported 171,578 malaria cases (cumulative incidence (CI) = 104 cases per 1,000 population) in 2009 and 221,244 malaria cases (CI = 119 cases per 1000 population) in 2010.

### Enhanced surveillance

To address challenges encountered within the HMIS and to improve the accuracy and consistency of data, an enhanced surveillance programme was initiated in all 26 public health facilities in Lusaka District beginning November 2010. The enhanced surveillance programme included four components: 1) retrospective record reviews of patient registers, tally sheets, and stock control cards to understand accuracy and process issues with reported malaria data; 2) prospective data collection using improved forms and reporting accountability measures at facility level; 3) improved quality assurance for diagnostic test results provided by facility-based laboratory technicians and the NMCC; and 4) consistent and timely reporting and feedback from the NMCC to the district and facility staff of results of prospectively collected information and quality assurance results.

Initially, retrospective malaria data were collected (January 2004 to March 2011) from each health facility in Lusaka District from a small team of data collectors. Due to high levels of incompleteness, only data starting from January 2009 were used in analyses. Data sources consulted included outpatient disease (OPD) registers, RDT registers, laboratory registers, stock control cards, and HMIS disease aggregation forms which are used to aggregate monthly facility data sent through the HMIS every month. Data collected included total consultations, total reported malaria, total malaria rapid diagnostic tests (RDTs) administered, total malaria microscopy slides collected, total confirmed malaria positives by RDT or microscopy, and total artemisinin combination therapy (ACT) courses dispensed. These data elements were adapted from a tool used by the PATH Malaria Control and Elimination Partnership in Africa (MACEPA) in other districts of Zambia [10]. The number of confirmed malaria cases reporting recent (within one month) travel history outside Lusaka District was also collected from clinic records starting in January 2012.

### Supervision and feedback

Between April 2011 and December 2012, the latest malaria data recorded in clinic logbooks was compared with reported numbers during monthly supervision visits, and feedback was provided to health facility staff on the previous month’s data. Clinicians, nurses, and laboratory staff were instructed on correct data definitions and reporting standards and were kept informed of their progress in adhering to national guidelines for treatment and reporting (e.g., dispensing anti-malarials only based on diagnostic confirmation). Areas in which improvements could be made were identified and corrected, such as use of case definitions or data recording practices. During monthly clinic visits, the data collection team provided reports to laboratory and clinic staff on their performance relative to other Lusaka District clinics. In addition, meetings between NMCP officers coordinating this activity and District Health Management Team (DHMT) staff were used to further disseminate information on the district malaria situation. These meetings brought the data to the forefront and helped foster an environment where the accuracy and quality of the data were considered important and helpful for decision-making.

### Laboratory quality assurance

The enhanced surveillance programme team partnered with the NMCP parasitology department to collect malaria laboratory quality assurance data. Each month all positive malaria blood slides as well as a sample of ten negative slides from each health facility were collected and re-examined by NMCP parasitology experts. RDT cassettes were also reviewed and compared to results, which clinic/lab staff had recorded. Week-long refresher training for all laboratory staff that included parasite detection, quantification, and *Plasmodium spp.* identification with WHO-approved reference slides was also provided. These trainings were conducted in May 2011 and included an additional component on malaria case management provided to at least one clinician from each health facility. These activities were all aimed at improving the accuracy of malaria diagnosis, case management, and ultimately, malaria surveillance.

### Data analysis

The benefits of enhanced surveillance activities were assessed through trend analysis using clinic-reported data collected during these activities. Four outcomes of principle interest were considered: the malaria test positivity rate, the malaria testing rate, the proportion of unconfirmed malaria, and costs associated with malaria diagnosis and ACT treatment. The malaria test positivity rate was defined as the number of malaria cases confirmed with RDTs or microscopy divided by the number of suspected malaria cases tested with RDTs or microscopy. The malaria testing rate was defined as the proportion of suspected malaria cases that were tested by either RDTs or microscopy. The proportion of unconfirmed malaria was defined as the number of reported malaria cases without diagnosis by RDT or microscopy divided by the number of total reported malaria cases. Median costs associated with malaria diagnosis were defined as $0.77 per 1,000 microscopy tests and $325 per 1,000 RDTs. Median costs associated with malaria treatment were defined as $800 per 1,000 ACT courses (NMCP personal communication). Differences in the outcomes before and after the implementation of enhanced surveillance were tested (2009–10 compared to 2011–12) by an interrupted time series analysis with health facility used as a random intercept. Total outpatient attendance and malaria transmission season were controlled by including monthly outpatient attendance and whether or not the month was part of the malaria transmission season which is assumed to be December – May. For the outcome of malaria test positivity, vector abundance was controlled for using the mean enhanced vegetation index (EVI) derived from MODIS satellites in a 2 km radius around each health centre. The relationship between enhanced surveillance data and routine HMIS was determined using Pearson’s correlation and a Z-test following Fisher’s Z transformation to compare correlation coefficients before and after implementation of enhanced surveillance. The Raster package [20] in R version 3.1.0 [21] was used to extract EVI for Lusaka District. All analyses were conducted using STATA 10.0 (College Station, TX), and R version 3.1.0 [21].

## Results

Data completeness of the enhanced surveillance programme exceeded 95 % for the study period. The enhanced surveillance system also allowed clinic data to be reviewed immediately upon monthly collection at the health facility, compared to a lag of three to six months or longer, which was common at that time within the routine HMIS system. Aggregated reported number of malaria cases in Lusaka District declined from 204,827 in 2009 to 130,374 in 2012 in conjunction with an actual decrease in the proportion of outpatients reported as malaria from 30.35 % in 2009 to 11.55 % in 2012 (Table 1).

**Table 1:** Total outpatient and malaria indicators reported through the enhanced surveillance programme in Lusaka, Zambia 2009–2012

Year	Total outpatient consultations	Reported malaria cases	Percent outpatient consultations reported as malaria	Mean testing rate	Mean test positivity
2009	674,824	204,827	30.35 %	18.94 %	6.41 %
2010	887,477	307,242	34.62 %	25.02 %	4.36 %
2011	911,230	209,945	23.04 %	51.04 %	4.26 %
2012	1,128,320	130,374	11.55 %	64.41 %	5.10 %

### Test positivity

**Fig. 1:**
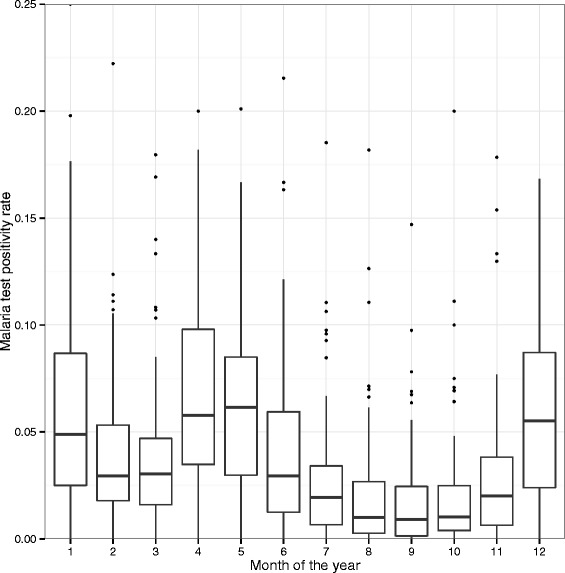
Range of malaria test positivity by month from 2009 to 2012 at all health facilities in Lusaka, Zambia. There were no differences in malaria test positivity by year

Test positivity at each facility rarely exceeded 10 % even in the high transmission season (Fig. 1). The odds of an individual testing positive for malaria were 2.86 times greater in the high transmission season than in the dry season (Odds Ratio (OR) = 2.86, 95 % CI = 2.30–3.56). The enhanced vegetation index was not associated with test positivity (Table 2). From 2009 to 2010, results suggest a moderate decreasing trend of test positivity with the odds of testing positive decreasing by 0.024 each month (*p* = 0.092). From 2011 to 2012 results suggest an increasing trend in test positivity with the odds of testing positive increasing by 0.042 each month (*p* = 0.026) (Table 2).

**Table 2:** Results from interrupted time series regression assessing the odds of testing positive for malaria in Lusaka District from 2009 to 2012. Standard errors have been adjusted for correlated data at the clinic level

	Factor	Odds ratio (95 % confidence interval)	*p*-value
Monthly trend	2009–2010	0.976 (0.949–1.004)	0.092
	2011–2012	1.042 (1.005–1.080)	0.026
Season	Dry	Reference	
	Wet	2.861 (2.297–3.564)	<0.001
Enhanced Vegetation Index	1st tertile	Reference	
	2nd tertile	1.157 (0.779–1.717)	0.454
	3rd tertile	0.943 (0.560–1.589)	0.820

*N* = 26 facilities and 548,158 suspected cases tested

### Improved testing rates

**Fig. 2:**
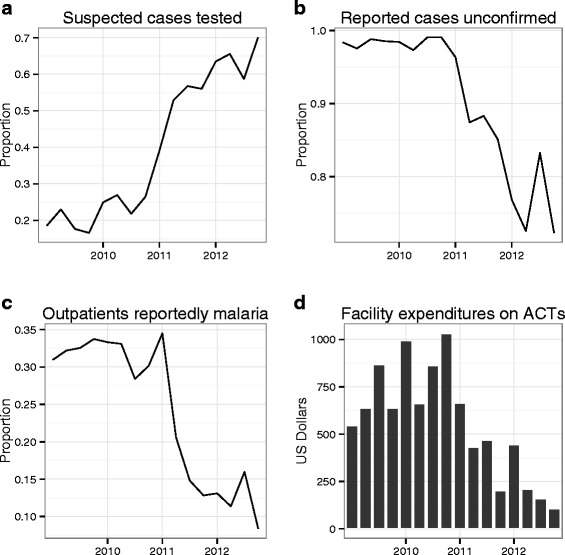
Clockwise from top left: **a** malaria testing rate, **b** proportion of reported malaria cases unconfirmed by microscopy or RDT, **c** proportion of total OPD that was reported as malaria, and **d** mean cost in ACT courses dispensed at each public clinic within Lusaka District from 2009 to 2012. Price of ACT in USD by pack size: 6′s = $0.36; 12′s = $0.72; 18′s = $1.08; 24′s = $1.30 (Medicine for Malaria Venture, USAID|Deliver, Zambia)

Before the implementation of the enhanced surveillance programme, 18.94 % of suspected malaria cases were tested with either microscopy or an RDT. Following the implementation of the enhanced surveillance programme, testing rates increased to 64.41 % with an immediate increase in January 2011 (Fig. 2a). The interrupted time series results suggest that the enhanced surveillance programme had a large impact on testing rates; the OR for a suspected malaria patient being tested was 1.54 (95 % CI = 0.96–2.49) following the introduction of the enhanced surveillance programme (Table 3). There was no trend in testing rates before the enhanced surveillance programme (*p* = 0.487), but following its introduction, the odds of receiving a diagnostic test increased to 1.048 per month (*p* = 0.023).

**Table 3:** Results from interrupted time series regression assessing the odds of suspected cases being tested for malaria in Lusaka District before and after the intervention of enhanced surveillance and feedback loop. Standard errors have been adjusted for correlated data at the clinic level

	Factor	Odds ratio (95 % confidence interval)	*p*-value
Time period	Pre-intervention	Reference	
	Post-intervention	1.543 (0.956–2.491)	0.074
Monthly trend	Pre-intervention	1.009 (0.983–1.036)	0.487
	Post-intervention	1.048 (1.007–1.091)	0.023
Season	Dry	Reference	
	Wet	0.974 (0.845–1.123)	0.708
Total outpatient attendance	Per 1,000	0.919 (0.883–0.956)	<0.001

*N* = 26 facilities and 1,345,978 suspected malaria cases

### Reporting confirmed malaria

In conjunction with the rising testing rates, the proportion of reported malaria that was unconfirmed by microscopy or RDT fell from 98.33 % before the implementation of the enhanced surveillance programme to 76.32 % after (Fig. 2b). The interrupted time series results suggest there was no change in the odds of a reported case being confirmed diagnostically before the enhanced surveillance programme (*p* = 0.511), and an increase of 0.090 per month in the odds of a reported case being confirmed following the intervention (*p* = 0.002). Reported malaria cases were more likely to be confirmed during the wet season (OR = 2.57, 95 % CI = 2.12–3.12) and with lower total outpatient attendance (Table 4).

**Table 4:** Results from interrupted time series regression assessing the odds of reported malaria cases being confirmed via rapid diagnostic test or microscopy for malaria in Lusaka District before and after the intervention of enhanced surveillance and feedback loop. Standard errors have been adjusted for correlated data at the clinic level

	Factor	Odds ratio (95 % confidence interval)	*p*-value
Intervention	Pre-intervention	Reference	
	Post-intervention	1.206 (0.367–3.962)	0.748
Monthly trend	Pre-intervention	0.987 (0.949–1.027)	0.511
	Post-intervention	1.090 (1.036–1.146)	0.002
Season	Dry	Reference	
	Wet	2.571 (2.122–3.116)	<0.001
Total outpatient attendance	Per 1,000	0.919 (0.867–0.975)	0.007

*N* = 26 facilities and 851,574 reported malaria cases

### Malaria-related costs

ACT consumption also decreased from 242,140 in 2010 to 119,790 in 2012. Lab diagnostic costs increased during this time period in conjunction with the testing rates. The interrupted time series results suggest that before the implementation of the enhanced surveillance programme, malaria costs were rising approximately 8.3 % per month (Table 5). Immediately following the implementation of the enhanced surveillance programme, malaria costs were higher than before the enhanced surveillance programme, but thereafter decreased 11.2 % each month.

**Table 5:** Results from interrupted time series regression assessing the monthly log-transformed costs associated with malaria testing and treatment in Lusaka District before and after the intervention of enhanced surveillance and feedback loop. Standard errors have been adjusted for correlated data at the clinic level

	Factor	Coefficient (95 % confidence interval)	*p*-value
Intervention	Pre-intervention	Reference	
	Post-intervention	1.398 (0.913 – 1.83)	<0.001
Monthly trend	Pre-intervention	0.083 (0.057 – 0.109)	<0.001
	Post-intervention	−0.112 (−0.147 – −0.077)	<0.001
Season	Dry	Reference	
	Wet	0.952 (0.752 – 1.151)	<0.001

*N* = 26 facilities and 1,236 facility-months

### Agreement between HMIS data and clinic records

**Fig. 3:**
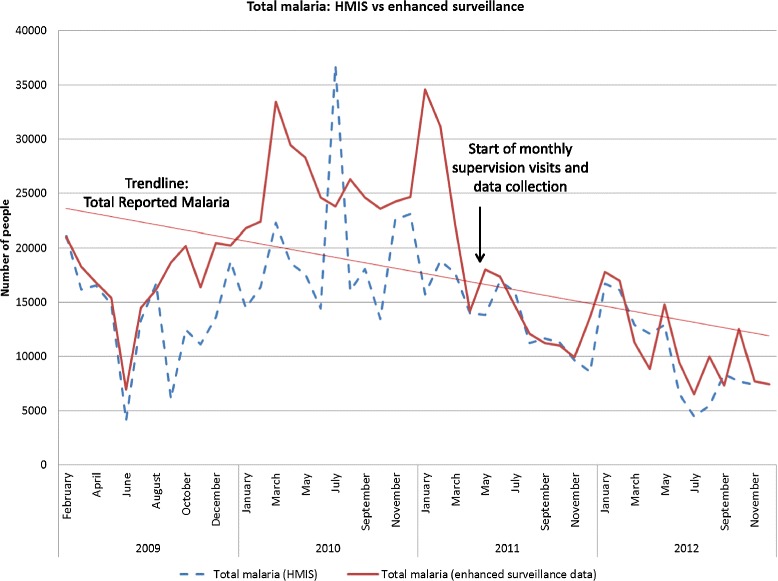
Comparison of total malaria cases reported through standard HMIS versus data collected through monthly supervision and review of clinic registers during the enhanced surveillance system (ES). Graph includes data from before and after the implementation of monthly enhanced surveillance system visits in 2011. Prior to the start of enhanced surveillance system, HMIS, and enhanced surveillance data were only moderately correlated compared to after, where correlation increased significantly

Prior to the initiation of enhanced surveillance, there was only a moderate correlation (total malaria, r = 0.476) between data recorded in clinic registers and the number of malaria cases actually reported to the HMIS (Fig. 3). During the enhanced surveillance period, these two datasets became not only more correlated (total malaria, r = 0.810), but the correlation increase was significant (z-score = −1.974, *p* = .048).

## Discussion

Clear benefits of the enhanced surveillance programme were observed during its implementation: a significant increase in the number of suspected cases being tested and a decrease in reported cases going unconfirmed. Costs associated with malaria treatment and diagnosis initially increased as testing rates increased with the implementation of the enhanced surveillance programme. However, the initial rise in cost was followed by a steady downward trend of 11.2 % per month. Prior to the enhanced surveillance programme, costs had been rising by 8.3 % per month. With 80 % of reported malaria still unconfirmed through diagnostics and a typical test positivity < 10 %, further cost savings can be obtained if more unconfirmed malaria cases are tested first and not given ACT if testing negative.

Between 2009 and 2012, total reported malaria cases fell considerably. The largest decline was seen in the number of clinical malaria cases, those thought to be malaria by the clinical staff but which did not receive a confirmatory malaria test. The decrease in clinical malaria cases likely do not represent an actual decrease in true malaria burden, as test positivity actually increased slightly. Rather, the enhanced surveillance programme likely contributed to increased confidence in the use of parasitological confirmations among patients by clinicians and increased perception among facility staff that malaria is not a significant problem in the district resulting in much fewer clinical malaria cases being reported.

Lack of diagnostic confirmation of malaria cases remains a major issue in Lusaka. In 2013, for example, 68 % of the 58,400 clinical cases reported in Lusaka District were reported from just 6 clinics (23 %). Greater outpatient attendance was associated with decreased odds of receiving a malaria diagnostic test and decreased odds that reported malaria was diagnostically confirmed. Among the clinics with persistently high rates of reporting unconfirmed malaria, the ability of facility staff to provide testing services to the number of patients seen remains a challenge. As the population growth of Lusaka outstrips the growth of more clinics and services, delays in receiving malaria diagnostics may increase. Where clinics struggle to offer testing services, screening at clinic registration, or shifting testing services to community level may be strategic solutions to improve malaria diagnostic and treatment rates.

Regular discussions with health facility staff during the feedback sessions influenced the perception of the malaria burden in their catchments. Prior to the initiation of the programme, health staff would comment on the high levels of malaria seen within their catchments; however, as the programme progressed, clinic staff directly observed data showing an increase in diagnostic use was associated with a decrease in unconfirmed reported malaria and the number of ACT dispensed. By the end of 2012, many staff recognised that the true burden of malaria was actually very low. It is worth noting that the laboratory training and mentorship provided likely contributed to the success of the feedback sessions, highlighting that a multi-partner framework drawing on specialist areas contributes to the success of surveillance programmes. Given this observed change, evidence-driven communication campaigns targeting health worker perceptions of malaria burden and the importance of diagnostic testing may be appropriate in reducing perceived malaria burden in other areas with similar malaria transmission and burden profiles as Lusaka district.

Despite testing rates exceeding 60 % following the implementation of enhanced surveillance, the proportion of malaria reported that was actually confirmed remained quite low at 24 %. In areas of low transmission such as in Lusaka, Zambia, with test positivity among suspected incident cases < 10 % throughout the year, unconfirmed reported malaria cases are not likely to be actual malaria cases. Misdiagnosing these individuals and giving them ACT presents two serious problems. First, in areas of low transmission such as Lusaka, the patient is likely not treated for the illness that prompted them to seek care. As an example of the problem of over prescription of ACT, in an area of Tanzania with a similar malaria test positivity rate as reported in Lusaka, respiratory infections treatable with antibiotics were the most common cause of fever among outpatients seeking care [22] Treating these individuals with an anti-malarial will be ineffective at best, but at worst may in fact increase morbidity and mortality [23]. Second, prescribing, and consuming drugs in the absence of the condition that they treat is a waste of money particularly egregious in resource-deprived settings. Understanding the aetiology and clinical diagnosis of non-malaria fevers is therefore a critical step in further reducing reported malaria and in improving patient care in Lusaka District.

Pressure to give a malaria diagnosis is multifaceted and includes a clinician’s treatment preferences, pressure from patients to receive medicine for an illness, availability of different medications, and the potential severity of missing a malaria infection [24]. Anecdotally, health practitioners in Lusaka District have indicated they are often pressured into prescribing anti-malarials in the absence of, or in spite of a negative diagnostic test result, by their patients. It is possible that overconsumption is a reflection of the paucity of treatments available for alternative diagnoses (e.g., antibiotics for respiratory infections). For example, when patients were provided with test results and alternative treatments in the case of negative malaria tests in Tanzania, patient pressure for anti-malarials reduced greatly [25]. A diagnostic algorithm requiring laboratory-confirmation for malaria infection before patients receive an anti-malarial has been implemented within Chelstone Clinic in Lusaka, resulting in a 65 % reduction in ACT courses dispensed between 2011 and 2012. Behaviour change communication campaigns to educate the community and urge patients to insist on receiving a diagnostic confirmation as well as modify the common perception that “fever equals malaria” could potentially help continue to reduce ACT over-consumption, as could behaviour change communication campaigns targeted toward clinicians.

Significant cost savings for the malaria control programme have likely already accrued as a result of the enhanced surveillance programme through reduced ACT dispensing. Despite this, the number of ACT courses dispensed continues to be much greater than the number of confirmed malaria cases. Further addressing the barriers to clinical reporting will improve the cost savings at the clinic level as well as at the district and NMCP levels. Additional benefit to the improved surveillance system has been a reduction in the allocation of other malaria resources to Lusaka District, in favour of more malarious areas as well as targeting of indoor residual spraying resources within Lusaka (2014). Targeting malaria resources based on improvements in surveillance ensures more efficient use of public and donor resources.

Given the low number of confirmed malaria cases identified, an additional component has been added to the enhanced surveillance programme: a reactive case detection system whereby confirmed cases are followed up to the household for foci detection and containment (Larsen et al. in preparation). This measure has been implemented to increase the sensitivity of the surveillance system to detect community-level malaria infections. Although Lusaka District has not been declared a malaria-elimination zone within Zambia, travel history, and other data necessary to substantiate malaria elimination are being collected in the hope that this area will soon report zero locally-acquired cases.

Progress towards both malaria control and eventual elimination are underpinned by high quality routine surveillance to determine the true malaria burden in an area. Further, in malaria control areas, accurate data are essential to know the overall burden of malaria as well as to identify hotspot areas so intervention resources can be applied wisely. The approach herein may be used in the short term to improve data capture and reporting practices, to increase adherence to best-practice policies, to inform local practitioners on the true burden of malaria in their area and to establish a robust foundation for the introduction of other interventions. Although the process of monthly supervision and data collection requires additional manpower to accomplish, local data clerks can be trained to systematically collect data, and over time health facilities will become accustomed to having record books available for monthly reviews. Further, in 2013, MOH clinics within Lusaka District as well as Southern, Central, and Western Province were transitioned to a system of weekly mobile-phone reporting of malaria data into the DHIS2, designed to support continued flow of accurate and timely malaria data [26]. Data transmitted include aspects of OPD attendance, clinical, and confirmed malaria case numbers, testing rates (RDT, and microscopy) and ACT consumption. The utilization of this new system has improved the timeliness of the data as well as provided a feedback loop to ensure clinic staff can visualize data trends. Staff at all levels of the health system are now able to login to the DHIS2 to view a dashboard displaying malaria data, which can highlight areas where specific clinics are achieving success in establishing high testing rates or reducing overconsumption of ACT. Through the dashboard, aspects that need to be addressed at individual clinics are visualized. Clinic staff can review malaria trends at their clinic, as well as in comparison with other clinics. Malaria data for the entire district, province, and nation can also be reviewed and supervisors at all levels can use this system as a supervisory tool. The rollout of weekly reporting and DHIS2 in Lusaka District was intended, in many ways, to replace the monthly supervision visits conducted during the enhanced surveillance system. Initial trends, however, indicate continued supervision at clinic level is necessary, at least in some clinics, to maintain the achievements in quality reporting made through this programme. Continued work to identify best-practice methods for ensuring health staff are reviewing and understanding data trends, and applying data knowledge to every-day clinical practice are being explored. These may include automated feedback mechanisms through DHIS2 and mobile phone technology to reduce the manpower required for consistent supervision visits. While private clinics were not included in this programme, public-private partnerships could be explored to ensure maximum data capture and harmonisation of best practices across the entire country.

## Conclusions

The enhanced surveillance programme initiated in Lusaka District achieved its objectives of improving case management and reporting of malaria. Most notably, the malaria testing rate increased substantially despite having further room for improvement. To ensure sustainability, and avoid the creation of a parallel system, key features of the programme, (e.g., feedback loops between clinic, and DHMT, and rapid reporting mechanisms) are now being incorporated into the HMIS via DHIS2. Systems such as the one described here may be worthwhile to maintain until improved data reporting practices take hold to determine the true burden of malaria in an area and to guide decision making on how to allocate limited malaria prevention and control resources.

## Abbreviations

ACT: Artemisinin Combination Therapy
CI: Cumulative Incidence
DHIS2: District Health Information System 2.0
GRZ: Government of the Republic of Zambia
HMIS: Health Management Information System
MACEPA: Malaria Control and Elimination Partnership in Africa
MIS: Malaria Indicator Survey
MOH: Ministry of Health
NMCP: National Malaria Control Programme
RDT: Rapid Diagnostic Test
